# Persistent Rhesus Enteric Calicivirus Infection in Recombinant CHO Cells Expressing the Coxsackie and Adenovirus Receptor

**DOI:** 10.3390/v16121849

**Published:** 2024-11-28

**Authors:** Tibor Farkas, Zeinab R. Aboezz

**Affiliations:** Department of Veterinary Pathobiology, College of Veterinary Medicine & Biomedical Sciences, Texas A&M University, College Station, TX 77843, USA; zeinab@tamu.edu

**Keywords:** recovirus, recombinant CHO cell, coxsackie and adenovirus receptor, persistent infection

## Abstract

Recently, using a panel of recombinant CHO cell lines, we identified the coxsackie and adenovirus receptor (CAR) and histo-blood group antigens (HBGAs) or sialic acid as the minimum requirement for susceptibility to rhesus enteric calicivirus (ReCV) infections. While ReCVs cause lytic infection in LLC-MK2 cells, recombinant CHO (rCHO) cell lines did not exhibit any morphological changes upon infection. To monitor infectious virus production, rCHO cell cultures had to be freeze–thawed and titrated on LLC-MK2 monolayers. This raised the question of whether ReCV infection in rCHO cells is persistent and whether non-enveloped progeny virions are released from the infected cells. Here, we used the rCHO-CAR+ cell line and a CAR and sialic acid-dependent recovirus strain (FT7) and found that these cells were persistently infected, and infectious virus was continuously produced and released into the culture without showing any visible cell damage. Viral capsid protein and replication intermediate double-stranded RNA (dsRNA) were detectable in almost all cells for at least 12 passages. We suspect a fully exosomal viral exit mechanism without a lytic cycle in these cells. rCHO cell may provide a valuable system for ReCV production (producer cell line) and serve as a model for investigating enteric calicivirus non-lytic viral exit mechanisms and the properties of the released, most likely membrane-cloaked, infectious progeny virions.

## 1. Introduction

The *Caliciviridae* family consists of a diverse group of small, non-enveloped viruses with a 7–8 kb positive-sense RNA genome. Among these, human noroviruses (HuNoVs) (genus: *Norovirus*) are the leading cause of viral acute gastroenteritis outbreaks globally [1]. Despite recent advances in cultivating HuNoVs in vitro, a simple and robust cell culture system for their continuous propagation is still lacking. To circumvent this bottleneck, several tissue culture-propagable caliciviruses have been used as surrogates for HuNoV research, including rhesus enteric caliciviruses (ReCVs) (genus: *Recovirus*). ReCVs exhibit remarkable biological similarities to HuNoVs, including histo-blood group antigen (HBGA) binding, genetic and antigenic diversity, and the manifestation of acute gastroenteritis in the host [2].

Recently, we identified the coxsackie and adenovirus receptor (CAR), a tight junction molecule, as the ReCV entry receptor [3]. In a follow-up study, we demonstrated that cell surface expression of CAR and HBGAs or sialic acid controls strain-specific susceptibility to infection [4]. During these studies, we created a panel of recombinant CHO (rCHO) cell lines expressing CAR and the type A, B, H HBGAs alone or in combination. While some of these cell lines became susceptible to ReCV infection and produced high titers of infectious virus, due to the lack of morphological changes in the infected cells, this could be detected only by qRT-PCR or titration in LLC-MK2 cells. The lack of lytic infection in rCHO cells was somewhat surprising since all ReCV strains studied in our laboratory caused lytic infection in LLC-MK2 cells, with visible cytopathic effects (CPEs), eventually destroying all the cells in the cultures within 4–5 days.

In the typical replication cycle, caliciviruses enter host cells through receptor-mediated endocytosis, followed by the release of their genomic RNA from the capsid to the cytoplasm. This RNA can act as mRNA (with a 5′-Vpg and 3′-poly-A tail) and hijacks the host translational machinery to initiate viral protein translation. The viral polyprotein is translated and self-cleaved by viral proteases into functional non-structural (NS) proteins, including the viral RNA-dependent RNA polymerase (RdRp). Viral NS-proteins and host proteins induce intracellular membrane remodeling for the assembly of viral replication complexes (virus factories), where negative-sense RNA is transcribed to serve as a template to produce genomic and subgenomic RNA (sgRNA). At this stage, the plus-sense genomic or sgRNA and the complementary negative-sense RNA can create replication intermediate dsRNA. Once enough structural proteins and genomic RNA are generated, new virions assemble and exit the cell [5].

Since non-enveloped viruses are not surrounded by a lipid membrane, they cannot exit the infected cell by membrane fusion, unlike most enveloped viruses. The mechanism of non-enveloped virus exit was thought to be through host cell lysis, involving rupturing the cell membrane at the end of the replication cycle and releasing the progeny virions. The cell with the compromised membrane would eventually die, and this process would be visible due to the morphological changes in the cell culture (CPE). Recent studies, however, have demonstrated that several non-enveloped viruses, including human noroviruses, exit infected cells before cell lysis via exocytosis as vesicle-cloaked virus clusters [5,6,7,8]. In these systems, however, exosomal release is eventually followed by a lytic phase and cell death.

Based on observations in our previous studies, we set out to investigate ReCV infections in rCHO cells and hereby report that these cells experience a productive, persistent infection, continuously producing high titers of infectious virus, released to the media without any visible evidence of cell damage.

## 2. Materials and Methods

### 2.1. Virus Strains and Cell Lines

The ReCV-FT7, Tulane virus, and ReCV-FT285 strains were used to infect the rCHO-CAR+ cell line, which was generated in our previous study [3]. The LLC-MK2 cell line was used for growing virus stocks and determine infectious virus titers. To prepare virus stocks, 80% confluent LLC-MK2 cell monolayers grown in tissue culture flasks were infected at an MOI of 0.1 and cultured for 48 h. At this point, most of the cells were rounded and detached. The flasks were subjected to 3 freeze–thaw cycles, and the contents were harvested, cleared by centrifugation, and stored at −80 °C in 1 mL aliquots. Both cell lines were grown in α-MEM medium supplemented with 10% fetal bovine serum (FBS) and penicillin/streptomycin/amphotericin B (P/S/A).

### 2.2. Virus Titration

LLC-MK2 cells (1 × 10^4^ cells/well) seeded in 96-well plates were inoculated with ten-fold serial dilutions of samples (100 µL/well, 3 wells/dilution). The plates were incubated for 5 days and stained with crystal violet. Virus titers (TCID_50_) were calculated using the Reed and Muench method.

### 2.3. rCHO-CAR+ Cell Infection and Passaging

rCHO-CAR+ cells were seeded onto 24-well plates and, at 80% confluency, overlayed with 250 µL culture media containing 1 MOI of the corresponding ReCV strain. The plates were incubated for 1 h at 37 °C, washed 3 times with media, and overlayed with 1 mL culture media/well. Six infected and uninfected control wells were included in each plate to monitor for possible cross-contamination during passages. The cultures were passaged every 3 days, at which point, the culture media were collected individually from 3 of the 6 infected and control wells for assessing the virus titers in the media. Debris was removed by centrifugation, and the samples were stored frozen at −80 °C. Once the media had been removed, the cells in these wells were trypsinized, and each well was passed to 2 fresh wells on a new plate at a 1:3 ratio. The plates with the remaining 3 wells containing the cultures were frozen and harvested after three freeze–thaw cycles to assess the total viral load.

### 2.4. Immunofluorescence

Uninfected and infected rCHO-CAR+ cells grown on chamber slides were fixed with 4% paraformaldehyde (PFA) and permeabilized with PBS-0.1% Triton X-100. Before dsRNA staining, half of the wells of the chamber slide were treated with RNase III (2U in 200 µL) for 3 h at 37 °C. Untreated wells were overlayed with 200 µL PBS during this time. The slides were washed with PBS-0.1% Tween 20 (PBS-T, pH 7.4) and blocked with PBS-T-3% BSA for 2 h at room temperature. All antibodies used were diluted in PBS-T-3% BSA. For viral protein detection (VP1), an in-house mouse serum generated by immunizing mice with purified ReCV-FT7 served as the primary antibody. For dsRNA detection, anti-dsRNA monoclonal antibody clone J2 (Scicions) was used. Both primary antibodies were applied at a 1:250 dilution. The slides with the primary antibodies were incubated overnight at 4 °C. Then, Alexa Fluor™ 594 labeled, F(ab’)2-Goat anti-Mouse IgG (H+L) Cross-Adsorbed Secondary Antibody (Invitrogen, Waltham, MA, USA, cat: A-11020) was used at a 1:2000 dilution for signal detection in both cases. The slides with the secondary antibodies were incubated for 1.5 h at RT in the dark. Between incubations, the slides were washed three times with PBS-T for 5 min. After the final wash, the chamber slides were disassembled and mounted using ProLong Diamond Antifade Mountant with DAPI (ThermoFisher Scientific, cat: P36962). The mounted slides were cured overnight in the dark at room temperature, and the edges were sealed with nail polish. Images were captured on an Evos FLoid^®^ Cell Imaging Station (ThermoFisher Scientific, Waltham, MA, USA).

### 2.5. Cell Viability

Cell viability was evaluated by trypan blue exclusion assay. The CellTiter-Glo^®^ 2.0 Luminescent Cell Viability assay (Promega, Madison, WI, USA) was used to evaluate metabolic activity in mock- and ReCV—FT7-infected CHO-CAR+ cells.

### 2.6. Statistical Analyses

Experiments were performed in at least three independent replicates. Differences between virus titers were evaluated for statistical significance by one-way ANOVA or t-test using the GraphPad software V6 (Dotmatics, Boston, MA, USA). A *p*-value ≤ 0.05 was considered statistically significant.

## 3. Results

### 3.1. ReCV Infection in rCHO Cell Lines Is Persistent and Non-Lytic

**Figure 1:**
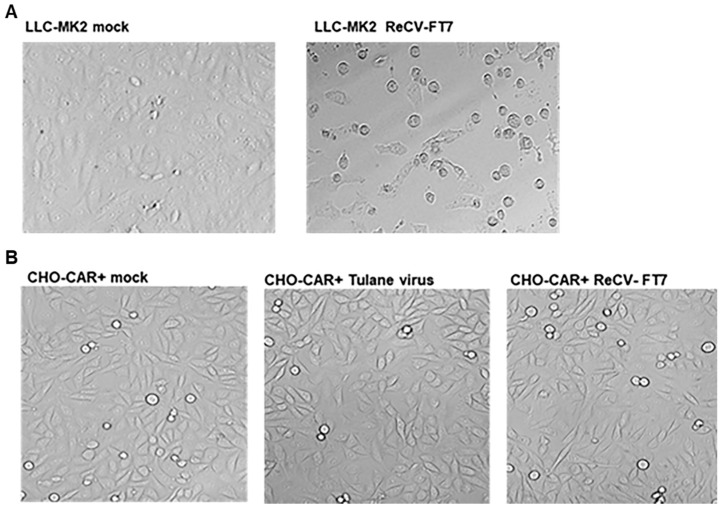
ReCV infection in rCHO cell lines remains non-lytic through serial passages. (**A**). ReCV infection can be seen in LLC-MK2 cells with characteristic CPEs of cell rounding and detachment. Cultures are shown 48 h post infection (0.1 MOI). (**B**). No morphological changes are observable between uninfected and infected rCHO cell cultures. Cell monolayers at passage 9 are shown.

**Figure 2:**
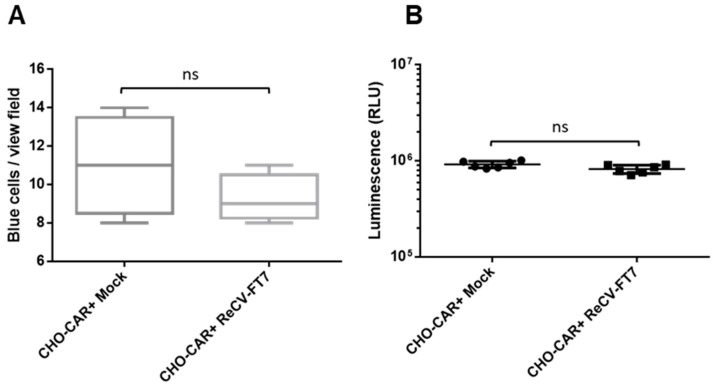
Cell viability and metabolic activity of mock- and ReCV-FT7-infected CHO-CAR+ cells. (**A**). Trypan blue exclusion assay results are shown, where the number of blues cells was counted in 5 view fields of mock- and ReCV-FT7-infected monolayers at passage 12 after 3 days of cell splitting. (**B**) Metabolic activity was measured at passage 12 after 3 days of cell splitting in 96-well white-walled tissue culture plates seeded with an equal number of cellsThe CellTiter-Glo 2.0 assay quantifying the amount of ATP was used according to the manufacturer’s protocol. The luminescence signal was recorded on a Cytation 5 multimode reader. Background-subtracted raw RLU values are shown. At least 3 wells were analyzed in 2 replicates. Means and standard deviations (SD) are graphed. Statistical significance was evaluated using a paired t-test; *p* ≤ 0.05 was considered statistically significant. ns: not significant.

While infection in LLC-MK2 cells is lytic with characteristic CPEs of cell rounding and detachment (Figure 1A), infection in rCHO-CAR+ cell monolayers did not produce any morphological changes (Figure 1) or changes in cell viability and cell growth (Figure 2A,B) for at least up to 12 passages.

### 3.2. Infectious Virus Production

**Figure 3:**
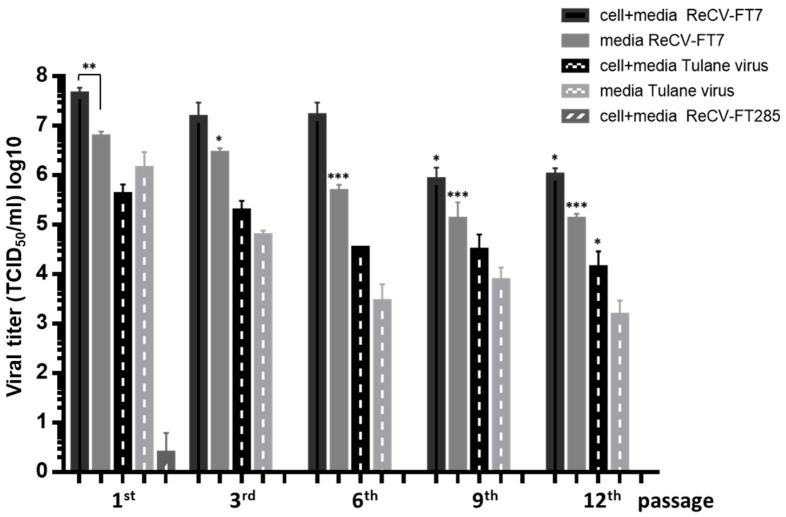
ReCV infection in rCHO cell lines is persistent, and infectious virus is efficiently released into the medium. rCHO-CAR+ cells were infected with ReCV-FT7, Tulane virus, and ReCV-FT285. Cultures were passaged every 3 days and examined for CPE. Total virus titers and cell-free virus titers were determined by titration in LLC-MK2 cells. Means and standard deviations (SDs) are graphed. Statistical significance was evaluated using the one-way ANOVA test with multiple comparisons. * *p* ≤ 0.05; ** *p* ≤ 0.01; and *** *p* ≤ 0.0001.

Infectious virus is continuously produced and released into the media. As expected, both ReCV-FT7 and Tulane viruses were able to infect and replicate in the rCHO-CAR+ cells, whereas ReCV-FT285, which relies on CAR and type A or B HBGAs for infection, was not able to infect this cell line [4] (Figure 3). Both ReCV-FT7 and Tulane virus-infected cultures produced infectious virus for at least up to 12 passages. The initial viral titers in the ReCV-FT7 infected cultures were almost 1 log higher than the titers of the virus stocks produced in LLC-MK2 cells (mean: 5.59 × 10^6^; SD: 1.88 × 10^6^ TCID_50_/mL). At later passages (≥9th), a statistically significant (1–1.5 log) drop was noticeable, bringing titers slightly below the titer of the virus stocks produced in LLC-MK2 cells. In the Tulane virus-infected cultures, starting from the first passage, virus titers were lower than the titer of the virus stocks grown in LLC-MK2 cells (mean: 4.57 × 10^6^; SD: 2.61 × 10^6^ TCID_50_/mL), which was expected. Our previous study demonstrated that, while Tulane virus can utilize both sialic acid and type A or B HBGAs, infection is more efficient in HBGA-expressing cells [4]. LLC-MK2 cells express type B HBGA, while, in rCHO-CAR+ cells, Tulane virus infection initiates through sialic acid attachment. A drop in titers towards the later passages was also noticeable in the Tulane virus-infected cultures. Importantly, in both cases, infectious virus was released into the culture media in high titers without any evidence of lytic infection. The virus titer in the media was slightly lower (0.2–07 log) than the titers in the corresponding whole-culture lysates and followed a similar pattern, with a slight drop towards the later passages (Figure 3).

### 3.3. Viral Protein and dsRNA in ReCV-Infected rCHO Cells

**Figure 4:**
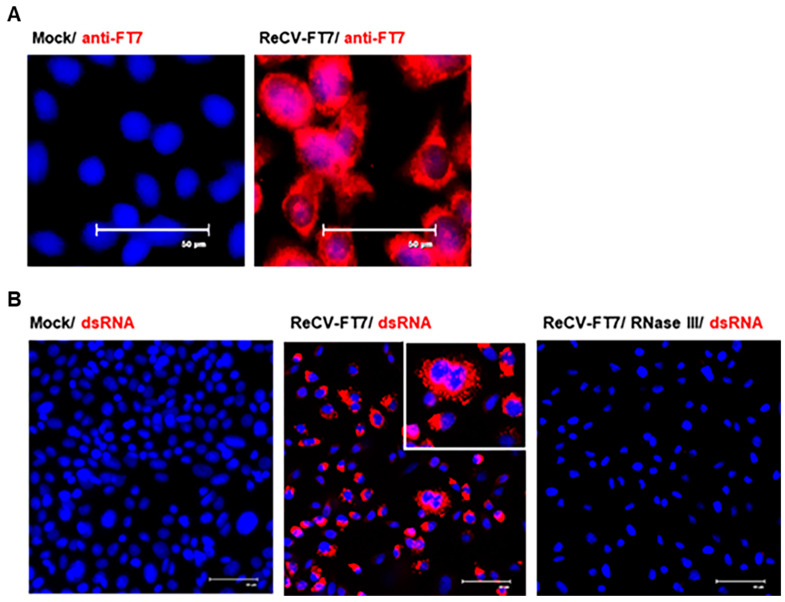
ReCV-infected rCHO cells contain viral protein and replication intermediate dsRNA. (**A**). Mock and infected rCHO-CAR+ cells were stained with mouse serum with serotype-specific VN activity against ReCV-FT7. (**B**). Mock and infected rCHO-CAR+ cells were stained with the dsRNA specific monoclonal antibody J2. Monolayers treated with RNase III were also included. Images represent cultures at passage 6 of the persistently infected cell line.

To further evaluate infection in rCHO-CAR+ cells, mock- and ReCV-FT7-infected monolayers were examined for the number of infected, virus-producing cells by immunostaining for VP1 (major capsid protein) and dsRNA. In both early and late passages, VP1 and dsRNA were detectable in the cytoplasm of almost every cell (five view fields/sample imaged) in the infected cultures (Figure 4A,B). No staining was detectable in the mock-infected cultures and secondary antibody controls. Furthermore, in the RNase III-treated monolayers, the fluorescence signal was reduced or completely void (Figure 4B). This signal reduction was RNase III concentration- and incubation time-dependent.

## 4. Discussion

Since the discovery and cell culture adaptation of the prototype Tulane virus [9], we have reported the detection and isolation of over 70 ReCV strains from rhesus macaques [10]. Despite using several parallel animal and human cell lines for the isolation of the Tulane virus, we were only able to detect the presence of viral RNA (RT-PCR) or observe any CPE in the LLC-MK2 cultures, and this was only after several blind passages [9]. We did not investigate the reasons behind the selective advantage in LLC-MK2 cells for ReCV isolation; however, most ReCV strains after isolation and passaging in LLC-MK2 cells were able to replicate in several other cell lines (e.g., Vero, MA104) (unpublished data). Indeed, in our genome-wide CRISPR library screening study which led to the identification of the ReCV entry receptor, we utilized a recombinant Vero cell line that constitutively expressed Cas9 [3]. The ReCV strains we tested caused lytic infection in all of these cell lines. A similar observation has been reported recently, where successful isolation of ReCV could be achieved only in LLC-MK2 cells, but virus stock produced later could be used to infect other non-human primate and even human cell lines [11]. This, however, was most likely not a special feature of the ReCV strain, as the title of the manuscript suggests, nor does it provide proof of the strain’s ability to infect humans. Most non-human primate (NHP) and human epithelial cell lines express ReCV susceptibility factors, and we have demonstrated that both rhesus and human CAR are sufficient to support ReCV infection in a cell culture. Other factors that influence in vitro cell tropism, such as those controlling permissiveness to ReCVs, remain to be discovered.

Most human cell lines replicated the ReCV poorly compared to the LLC-MK2 or Vero cells, and only the two NHP cell lines exhibited CPE and robust virus replication. The authors did not provide data on whether the infections in the human cell lines were lytic or not, and whether the lack of visible CPE was due to the low efficiency of infection (low number of infected cells which went through lytic infection). Regardless, to the best of our knowledge, until now, all reported in vitro ReCV infections producing high virus titers have been lytic.

Wild-type CHO cells are resistant to ReCV infection. This is due to a lack of susceptibility (lack of CAR and HBGA expression), but they are permissive (able to support steps of replication cycle after entry). This made them the cell line of choice for our recent studies aiming to better understand the host requirements for susceptibility to ReCV infections [3,4]. We created a panel of rCHO cell lines expressing cell-surface CAR and type A, B, or H HBGAs [3,4]. Wild-type CHO cells not only lack CAR and HBGA expression but are also deficient in functional α2,6-sialyltransferase (ST6GAL1) and have incomplete sialylation, with only α2,3-linked sialic acid [12]. While several of the rCHO cell lines expressing CAR and HBGAs became susceptible to ReCV infections and produced high titers of infectious virus, none of these infections were lytic or led to any visible morphological changes [4]. We only needed to observe one round of infection in our previous study and evaluated the whole-cell lysate for virus production to establish the gain of susceptibility.

The goal of the current study was to further investigate the seemingly non-lytic infection in rCHO cells and determine whether it was a persistent infection or transitioned to an abortive infection at later passages and whether the progeny virus was still released from the cells showing no evidence of membrane damage. We demonstrated that ReCV infection in rCHO cells is a productive, persistent infection without a lytic cycle (Figure 1). We monitored persistently infected and control rCHO-CAR+ cells during 12 consecutive passages over about 5 weeks. ReCV-FT7- and Tulane virus-infected cultures, but not mock- or ReCV-FT285-infected cultures, continuously produced high viral titers, and infectious virus was released from the cells into the media (Figure 3). Immunofluorescence staining demonstrated that almost all cells were filled with viral capsid protein and replication intermediate dsRNA (Figure 4A,B). Interestingly, ReCV-infected rCHO-CAR+ cells did not show any changes in viability or metabolic activity compared to mock-infected cells (Figure 2A,B). However, there was an evident drop in virus titers with increasing passages of the persistently infected cultures. Identifying the reason behind this will need further investigations, but a similar fluctuation in virus titers in persistently infected cell cultures has been described previously, which could be explained by the production of defective interfering particles (DIPs), changes in the physiological state of the host cells, and periodic changes in viral immune evasion mechanisms or environmental conditions [13,14].

Productive, persistent infection in cell cultures has been reported for many viruses [15,16]. While persistent calicivirus infection has been reported in immunocompromised patients (HuNoV), mice (MNV), and cats (FCV), to the best of our knowledge, this is the first report describing cell line-dependent persistent calicivirus infection. Further characterization is necessary to address the following: (1) the survival mechanism of persistently infected CHO cells producing large amounts of viral proteins and dsRNA; (2) if there is viral immune evasion circumventing the detection of viral products, especially the large amounts of dsRNA by cytosolic PRRs (e.g., RIG-I, MDA5, PKR, NLRP1); and (3) the mechanism of viral exit through intact cell membranes (e.g., exosomes, vesicle-cloaked virus clusters). Nonetheless, our study suggests that the rCHO cell lines generated in our laboratory could serve as a cell line for producing high-titer infectious ReCV stocks and as a research model to study all the above-mentioned areas. We are particularly interested in the viral exit mechanism. Moreover, we hope that future studies using this system will also yield important information for human norovirus research.

## Data Availability

Data available upon request.
